# Association Between Subjective Memory Complaints in Daily Life and Smartphone Proficiency Among Community-Dwelling Middle-Aged and Older Adults: A Cross-Sectional Population-Based Study

**DOI:** 10.7759/cureus.72578

**Published:** 2024-10-28

**Authors:** Suguru Shimokihara, Yuriko Ikeda, Fumiyo Matsuda, Takayuki Tabira

**Affiliations:** 1 Research Fellowship for Young Scientists, Japan Society for the Promotion of Science, Tokyo, JPN; 2 Department of Occupational Therapy, School of Health Sciences, Sapporo Medical University, Sapporo, JPN; 3 Faculty of Medicine, Kagoshima University, Kagoshima, JPN; 4 Department of Occupational Therapy, School of Health Sciences, Faculty of Medicine, Kagoshima University, Kagoshima, JPN; 5 Department of Physical Therapy, School of Health Sciences, Faculty of Medicine, Kagoshima University, Kagoshima, JPN

**Keywords:** community based, proficiency, smartphone use, subjective memory complaints, technology

## Abstract

Objective

The study aims to investigate the association between subjective memory complaints (SMCs) in the daily lives and smartphone proficiency of community-dwelling middle-aged and older adults.

Methods

This cross-sectional study used a self-administered questionnaire. Participants' SMCs were assessed with seven questions related to daily lives. Smartphone proficiency was evaluated using a short version of the Mobile Device Proficiency Questionnaire (MDPQ-16). Multivariate logistic regression models were used to explore the relationship between SMCs and smartphone proficiency, adjusting for covariates. Additionally, SMCs that were significantly associated with smartphone proficiency were compared by MDPQ-16 subcategory scores.

Results

A total of 676 participants aged ≥60 years (69.4±6.2 years, 87.0% female) were analyzed. Participants had the highest proportion of SMC for "forgetting names of friends or relatives" (67.9%). SMCs that showed significant associations with smartphone proficiency were "forgetting appointments and tasks" (OR: 0.98, 95% CI: 0.95-0.99, *p* = .044) and "forgetting the date" (OR: 0.96, 95% CI: 0.93-0.99, *p* = .007). Among the subcategories of the MDPQ-16, mobile device basics, communication, Internet, calendar, and entertainment were significantly lower in the group with SMCs (*p* <, .05).

Conclusion

Middle-aged and older adults with higher smartphone proficiency had significantly lower odds of "forgetting appointments and tasks" and "forgetting the date" among SMCs in daily life and they were more proficient not only in basic smartphone operation but also in various functions. In today's digital society, healthcare professionals may need to pay attention to smartphone proficiency to cover SMCs in daily life.

## Introduction

As the population ages around the world, achieving healthy longevity becomes increasingly important [1]. Improvements in medicine and nutrition continue to increase life expectancy [2,3], but maintaining cognitive function and quality of life for older adults is a current challenge. In particular, coordinating sustainable living for older adults in their own neighborhoods is of paramount concern to community healthcare professionals [4,5].

Maintaining and improving cognitive function is essential for a long and healthy life. Many studies of cognitive decline have shown that cognitive function progresses from normal to mild cognitive impairment and then to dementia [6,7], but subjective memory complaints (SMCs) have also been suggested as a preclinical stage of these disorders [8]. SMCs are the perception of a decline in memory function, regardless of the presence or absence of objective memory impairment. Rare instances of forgetting things, names, and appointments are usually within the normal range of aging [9]. However, when such things happen frequently, they are anxious about their memory condition [10]. SMCs have been shown to be associated with anxiety and depressive symptoms rather than objective cognitive function [11], which may have implications for mental health. Therefore, prevention and early intervention of SMCs in the preclinical period may prevent later poor outcomes such as the development of dementia and worsening mental health. In the *Diagnostic and Statistical Manual of Mental Disorders: 5th Edition *[12], one of the most commonly used diagnostic criteria for dementia in many countries today, the main point of diagnosis is that the disorder interferes with daily and/or social life and interpersonal relationships due to impairment in one or more cognitive domains. Therefore, reducing SMCs in daily life may prevent the decline in daily and social life impairment associated with a diagnosis of dementia and psychological status.

In recent years, smartphones have become increasingly popular among middle-aged and older adults as well as the younger population. Smartphones are expected to serve not only as a means of communication and information access but also as an aid for various tasks in daily life. Especially for older adults, smartphones enable convenient information retrieval, physical condition management, and communication. Previous studies have reported associations between smartphone use and several health-related outcomes in older adults. Smartphone ownership is known to be associated with higher cognitive function [13,14] and lower depressive symptoms [15] in older adults. It has also been reported to be associated with the absence of frailty [16,17]. Despite the interesting findings focusing on smartphone use and health status among older adults, a limitation of these reports is that they do not assess smartphone proficiency. This means that even if they are not proficient in using smartphones, they may still be classified as smartphone owners. This may lead to erroneous conclusions when considering the use of digital devices to support the health of middle-aged and older adults. The few studies that have examined smartphone proficiency and health have shown that it is associated with higher life competencies and physical function among older adults [18], but the association with SMC remains unclear. Being proficient in the use of smartphones is one possible solution to help people avoid potential SMC situations in their daily lives, and could be a means of supporting and compensating for the user's subjective memory.

We argue that in today's digitized society, smartphone proficiency should also be examined in detail to address daily life issues faced by middle-aged and older adults. Accordingly, we focused our attention on the association between smartphone proficiency and SMCs. We hypothesize that there is a relationship between high smartphone proficiency and the absence of SMCs in daily life among middle-aged and older adults. This study aims to explore the relationship between subjective memory complaints (SMCs) in daily life and smartphone proficiency among middle-aged and older adults.

## Materials and methods

Sample size calculation

To achieve our aim of determining the association between SMC and mobile device proficiency in a community-based observational study, we conducted a simulation of the required sample size. The prevalence of SMC in the general population is estimated to be approximately 25-50% [19], and previous simulation studies have shown that the minimum number of events in logistic regression analysis is not problematic for explanatory variables ×10 or greater [20]. Therefore, assuming the lowest prevalence in the study population (25%) and considering the five independent variables used in this study, we concluded that a minimum of 200 participants would be required.

Study design and participants

We used a self-administered questionnaire in a cross-sectional study (see Appendices). The questionnaire was designed to be user-friendly and self-explanatory, allowing participants to complete it on their own. Instructions for completing the questionnaire were provided in writing, and participants were encouraged to reach out if they had any questions or required further clarification. This research was carried out in the southern Japanese prefecture of Kagoshima. The inclusion criteria for participants were members of a consumer's cooperative (CO-OP) in Kagoshima, Japan, aged 50 years or older. 3,000 randomly selected members of the cooperative were sent a self-administered questionnaire inviting them to participate in the study. At the beginning of the questionnaire, participants were asked, "What type of mobile device(s) do you usually use?" Participants selected the appropriate one from a smartphone, flip phone, and personal computer. For the purposes of our study, we excluded those under the age of 60, those with incomplete responses, and those who did not use smartphones. CO-OP is a type of business owned and operated by its customers for their mutual benefit [21].

CO-OPs prioritize member needs over maximizing profits, fostering community engagement and democratic decision-making. They often focus on sustainable practices, ethical sourcing, and local empowerment. Consumer cooperatives aim to enhance economic equity, promote social responsibility, and provide quality products or services to their members. CO-OP Kagoshima is a community-based organization that supports various community activities such as the sale of groceries and daily necessities through physical stores and home delivery. In 2020, the organization had 320,000 members, who provide financial assistance through their investments. In order to participate in this survey, invitees can either scan a QR code to answer online or complete the questionnaire and mail it back in an envelope without their address on it. Only the address of the researcher's institution was written on the envelope and the postage was paid by the researcher. The survey was carried out starting in November 2022 and lasting for one month. 1189 replies were received (39.6% response rate; 97.9% via mail). The study was conducted according to the guidelines of the Declaration of Helsinki and approved by the Ethics Committee on Epidemiological Studies, Kagoshima University (Ref No. 220071).

Operational definition of SMC

Various methods have been developed previously to assess SMCs [22-25]. In this study, the following seven questions about situations in daily life were used as subjective memory questions: (1) “Do you forget the names of close friends or relatives?”, (2) “Do you forget your appointment or task?”, (3) “Do you have difficulty remembering where you leave objects like a wallet or a key?”, (4) ”Do you have trouble figuring out what today’s date is?”, (5) ”Do you forget what you want to buy at the supermarket or other places?”, (6) “Do you forget your way around the neighborhood?”, and (7) “Do you consider yourself as being forgetful?”. Responses were recorded using the following four-subject format: often, sometimes, not often, and completely not. We operationally identified participants who answered "often" or "sometimes" for each of the seven questions as SMCs.

Assessment of smartphone proficiency

We used a short version of the Mobile Device Proficiency Questionnaire (MDPQ-16) [26] to assess smartphone proficiency among participants. This assessment evaluated participants’ proficiency in eight subitems related to mobile devices including smartphones, such as basics of a mobile device, communication, data and file storage, internet, calendar, entertainment, privacy, troubleshooting, and software management. The MDPQ-16 uses a 5-point Likert scale (1 = never tried, 2 = not at all, 3 = not very easily, 4 = somewhat easily, and 5 = very easily), and the questionnaire was scored as previously reported [26,27].

Demographical data

We obtained the demographic characteristics of the participants as a potential confounding factor, such as age, sex, years of education, and living situation (living alone or living with others). Age and years of education were used as continuous variables, while sex and living situation were used as categorical variables.

Statistical analysis

Participant characteristics were tabulated using descriptive statistics. Means and standard deviations (SD) were used for continuous variables, and the number of observations and percentages were used for categorical variables. We then used logistic regression analysis as a multivariate analysis to examine in detail the relationship between SMCs and smartphone proficiency. The details of the regression model are as follows: dependent variable; presence of SMCs (1 = presence of SMC), independent variable; MDPQ-16 score, covariates; age, gender, and living situation. Finally, SMC items that were found to be significantly associated with smartphone proficiency were explored to determine which areas of smartphone operation differed. scores on the bottom eight categories of the MDPQ-16 were compared using Welch's t-test in groups classified by the presence of SMC. Because the purpose of the test here was to explore associations, no correction for alpha error due to multiplicity was performed [28]. All statistical analyses were performed using R ver. 4.2.2 (R Foundation for Statistical Computing, Vienna, Austria), and the significance level was set at p < 0.05.

## Results

Characteristics of participants

The characteristics of the participants are shown in Table 1. A total of 676 participants were included in the analysis, with a mean age ± SD of 69.4 ± 6.2 years (female; n = 583, 87.01%). The most common SMC in daily life was forgetting names (67.9%), and the least common was forgetting way directions (1.5%).

**Table 1:** Characteristics of study participants. Count data are represented as n and %, and numerical data as mean and standard deviation. Abbreviations: MDPQ-16, short version of the Mobile Device Proficiency Questionnaire; SMC, subjective memory complaints. Scores on the MDPQ-16 range from 8-40.

Overall (n = 676)	Mean^*^ or n^#^	Standard deviation or %
Age, years	69.43^*^	6.15
Education years, years	12.88^*^	2.00
Sex (female), n	583^#^	87.01 %
Living situation (living alone), n	107^#^	15.99 %
MDPQ-16, score	22.61^*^	8.43
SMCs in daily life, n		
Forgetting the name of close friends or relatives	458^#^	67.85 %
Forgetting appointments and tasks	199^#^	29.44 %
Forgetting where to place something	218^#^	32.30 %
Forgetting the date	107^#^	15.85 %
Forgetting what things to buy	342^#^	50.82 %
Forgetting way directions of neighborhood	10^#^	1.49 %
Consider oneself forgetful	393^#^	58.14 %

Association between SMCs and smartphone proficiency

**Figure 1:**
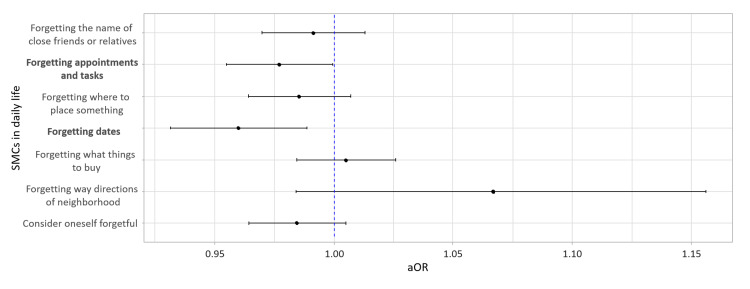
Adjusted odds ratios and 95% CIs of smartphone proficiency for each SMC. In each logistic regression model, the dependent variable is the presence or absence of SMC (reference: absence of SMC), the independent variable is the MDPQ-16 score, and the covariates are age, sex, and living status. Variables with p < .05 are in bold. Abbreviations: aOR, adjusted odds ratio; CI, confidential interval; MDPQ-16, short version of the Mobile Device Proficiency Questionnaire; SMCs, subjective memory complaints.

Figure 1 plots the odds ratios of the MDPQ-16 scores in a model with seven SMCs items as dependent variables. Detailed values for all models are presented in Table 2. In a logistic regression model to examine the association between SMCs and smartphone proficiency, smartphone proficiency was significantly associated with forgetting appointments (odds ratio; OR 0.98, 95% CI 0.95-0.99, p = .04) and forgetting dates (OR 0.96, 95% CI 0.93-0.99, p = .007). Both results indicate that higher smartphone proficiency was associated with lower odds of having SMCs.

**Table 2:** Details of 7 logistic regression models about SMC in daily living and smartphone proficiency of middle-aged and older participants. Abbreviations: CI, confidence interval; MDPQ-16, short version of the Mobile Device Proficiency Questionnaire; OR, odds ratio; SE, standardized error; SMC, subjective memory complaints.

SMCs in daily life	Variables	Estimate	SE	OR	95 % CI		p-value
					upper	lower	
Forgetting the name of close friends or relatives	Age	0.00	0.02	1.00	0.97	1.03	0.80
	Sex (ref: female)	0.11	0.25	1.11	0.68	1.83	0.67
	Education year	-0.04	0.04	0.96	0.88	1.05	0.34
	Living situation (ref: living with others)	0.20	0.24	1.22	0.76	1.95	0.41
	MDPQ-16 (score)	-0.01	0.01	0.99	0.97	1.01	0.42
Forgetting appointments and tasks	Age	0.00	0.02	1.00	0.97	1.03	0.81
	Sex (ref: female)	-0.81	0.25	0.45	0.27	0.73	0.00
	Education year	-0.02	0.05	0.98	0.89	1.07	0.59
	Living situation (ref: living with others)	-0.09	0.24	0.92	0.57	1.48	0.72
	MDPQ-16 (score)	-0.02	0.01	0.98	0.95	0.99	0.04
Forgetting where to place something	Age	-0.02	0.02	0.98	0.95	1.01	0.21
	Sex (ref: female)	-0.05	0.26	0.95	0.57	1.58	0.85
	Education year	-0.05	0.04	0.95	0.87	1.04	0.28
	Living situation (ref: living with others)	0.35	0.23	1.42	0.90	2.22	0.13
	MDPQ-16 (score)	-0.01	0.01	0.99	0.96	1.01	0.19
Forgetting the date	Age	0.01	0.02	1.01	0.97	1.04	0.77
	Sex (ref: female)	-0.17	0.32	0.84	0.45	1.57	0.59
	Education year	0.08	0.06	1.09	0.97	1.21	0.14
	Living situation (ref: living with others)	-0.40	0.33	0.67	0.35	1.27	0.22
	MDPQ-16 (score)	-0.04	0.02	0.96	0.93	0.99	0.01
Forgetting what things to buy	Age	-0.03	0.01	0.97	0.94	0.99	0.02
	Sex (ref: female)	0.56	0.25	1.76	1.09	2.85	0.02
	Education year	-0.06	0.04	0.95	0.87	1.03	0.19
	Living situation (ref: living with others)	0.09	0.22	1.09	0.71	1.69	0.70
	MDPQ-16 (score)	0.00	0.01	1.00	0.98	1.03	0.64
Forgetting way directions of neighborhood	Age	0.01	0.06	1.01	0.90	1.13	0.90
	Sex (ref: female)	0.46	1.09	1.59	0.19	13.49	0.67
	Education year	-0.02	0.17	0.98	0.71	1.36	0.90
	Living situation (ref: living with others)	0.27	0.83	1.31	0.26	6.64	0.74
	MDPQ-16 (score)	0.06	0.04	1.07	0.98	1.16	0.12
Consider oneself forgetful	Age	-0.01	0.01	0.99	0.96	1.02	0.55
	Sex (ref: female)	0.02	0.24	1.02	0.63	1.64	0.94
	Education year	-0.03	0.04	0.97	0.90	1.06	0.53
	Living situation (ref: living with others)	0.19	0.23	1.21	0.78	1.88	0.40
	MDPQ-16 (score)	-0.02	0.01	0.98	0.96	1.01	0.14

Exploration of differences in smartphone proficiency with and without SMC

Among the SMCs, we compared the eight subcategories of the MDPQ-16 on the items of forgetting appointments and forgetting dates, which were found to be significantly associated with smartphone proficiency. In Table 3 and Table 4, we present the means and statistics for each subcategory of the MDPQ-16 with and without SMCs in daily living. In the group of forgetting appointments, the scores for the basic operation (effect size (ES) = 0.32) and communication (ES = 0.21) categories were significantly lower. The group of date forgetting also had significantly lower scores on the basic operations (ES = 0.36), communication (ES = 0.39), Internet (ES = 0.26), calendar (ES = 0.24), and entertainment (ES = 0.21) categories.

**Table 3:** Table 2. Differences in mobile device proficiency with and without SMC (forgetting appointments and tasks) in daily living. SMC +/- indicates a group in which the respective SMC is present/absent. The values indicate the mean (standard deviation). The statistical analysis performed was Welch's t-test. Abbreviations: MDPQ-16, short version of the Mobile Device Proficiency Questionnaire; SMC, subjective memory complaints.

		SMC (+), n = 199	SMC (-), n = 477	Statistic	p-value	Effect Size (Cohen's d)
Forgetting appointments and tasks	MDPQ-16 subcategories					
	Mobile device basics	3.38 (1.25)	3.76 (1.11)	3.68	< .001	0.32
	Communication	3.81 (1.08)	4.04 (1.02)	2.51	0.01	0.21
	Data and file storage	2.31 (1.49)	2.37 (1.56)	0.42	0.68	0.03
	Internet	3.03 (1.47)	3.23 (1.54)	1.62	0.11	0.14
	Calendar	2.49 (1.54)	2.65 (1.68)	1.16	0.25	0.10
	Entertainment	2.11 (1.28)	2.22 (1.35)	1.01	0.31	0.08
	Privacy	2.37 (1.3)	2.56 (1.43)	1.64	0.10	0.14
	Troubleshooting and software management	2.09 (1.35)	2.21 (1.54)	1.04	0.30	0.09

**Table 4:** Differences in mobile device proficiency with and without SMC (forgetting the date) in daily living. SMC +/- indicates a group in which the respective SMC is present/absent. The values indicate the mean (standard deviation). The statistical analysis performed was Welch's t-test. Abbreviations: MDPQ-16, short version of the Mobile Device Proficiency Questionnaire; SMC, subjective memory complaints.

		SMC (+), n = 107	SMC (-), n = 569	Statistic	p-value	Effect Size (Cohen's d)
Forgetting the date	MDPQ-16 subcategories					
	Mobile device basics	3.29 (1.3)	3.72 (1.12)	3.21	0.002	0.36
	Communication	3.60 (1.2)	4.04 (1)	3.49	< .001	0.39
	Data and file storage	2.12 (1.46)	2.40 (1.55)	1.76	0.08	0.18
	Internet	2.84 (1.49)	3.23 (1.52)	2.50	0.01	0.26
	Calendar	2.27 (1.5)	2.66 (1.66)	2.40	0.02	0.24
	Entertainment	1.96 (1.22)	2.23 (1.34)	2.07	0.04	0.21
	Privacy	2.37 (1.36)	2.52 (1.4)	1.02	0.31	0.11
	Troubleshooting and software management	2.10 (1.43)	2.19 (1.49)	0.55	0.58	0.06

## Discussion

The prevalence of SMCs in older adults ranges widely from 11.7% to 84.2% [19,29,30]. One reason for this may be that SMC is self-reported. In other words, the results may vary depending on the situation of individuals at the time [31]. In fact, approximately 12% of middle-aged and older adults report different SMC status at each measurement [32]. Our results also show that the percentage of respondents with SMC varied greatly from question to question, ranging from 67.9% to 1.5%, although we prepared questions for a variety of situations related to daily life. In particular, the high prevalence of name forgetting can be a barrier to social interaction in the community. Previous studies have shown that people with SMC report low self-efficacy and inadequate social interactions [33]. Specifically, the anxiety of not being able to remember the names of friends or relatives may lead to restrained conversation and non-participation in greetings. Therefore, it is important to find a way to easily recall the name and ensure smooth communication prior to beginning SMC.

Of the seven SMCs in daily life, "forgetting appointments" and "forgetting dates" were significantly associated with smartphone use. Numerous studies have examined the effectiveness of memory support devices for older adults, but high-quality evidence is scarce [34]. The use of smartphones is likely to be effective for both types of SMCs. This is because many smartphones have calendars and to-do lists as standard features, making them easy to access. Future research should examine the effectiveness of smartphone use for SMC using randomized controlled trials or other high-level evidence methods. However, it should be noted that in our results, some SMCs were not significantly related to smartphone proficiency. For example, there was no relationship between SMCs and smartphone proficiency despite the possibility of using the smartphone's memo function for forgetting what to buy and navigation function for forgetting directions. It has now been suggested that it is inappropriate for assistive devices to be beyond the skill level of the user [35,36]. In other words, the features of smartphones may be too complex for middle-aged and older adults and they may not be able to fully use these features. Our results showed that participants with the SMC of forgetting appointments and dates scored significantly lower on the MDPQ-16 categories of smartphone use: basic use, communication, Internet, calendar, and entertainment. In other words, older adults may need support not only to own a smartphone but also to improve their smartphone proficiency from an early stage and to use it as an assistive device in their daily lives.

The strengths of our study lie in several key aspects. First, the large sample size allows for greater generalizability of the findings to the broader population of community-dwelling middle-aged and older adults. This represents a significant advantage, as it helps to ensure that the study's conclusions are applicable to this population, which is increasingly relevant in aging societies. Additionally, the use of validated instruments such as the MDPQ-16 strengthens the study's methodological rigor. Moreover, our study addresses an important issue by focusing on smartphone proficiency and its relationship with SMCs among middle-aged and older adults. This is a critical area of research, as smartphone use has become an integral part of daily life and social participation. Our findings provide valuable insights into how perceived memory problems may relate to technology use in older age, potentially informing interventions aimed at improving both technology adoption and cognitive health. The study thus contributes meaningfully to the ongoing discussion about supporting older adults in maintaining functional independence through technological means.

However, several limitations must be recognized. First, as a cross-sectional study, it is difficult to mention the causal relationship between SMC and smartphone proficiency. A follow-up study will be conducted to further clarify the relationship between SMC and smartphone proficiency. Second, the selection bias of self-administered questionnaires has not been completely eliminated; the presence of SMC often depends on the cognitive function and psychological state of the participants [11,37]. Because the participants in this study were limited to those who were able to complete and return the questionnaires, it is possible that the participants were limited to those with better cognitive and mental function. Future research should include a brief objective assessment of cognitive and mental status. Finally, a variable related to economic status, which could be a potential confounding factor related to smartphone ownership, is missing. It is possible that participants with lower incomes also have lower rates of smartphone ownership, a point that needs to be clarified in future studies. Even with these limitations, our study, which clarified the relationship between SMCs in the daily lives and smartphone proficiency of middle-aged and older adults, is expected to provide important foundational knowledge to support people living in a digital society.

## Conclusions

In this study, we examined the relationship between SMCs in daily life and smartphone proficiency among 676 community-dwelling middle-aged and older adults. The results showed that participants with high smartphone proficiency had significantly lower odds of the presence of SMCs in "forgetting appointments and tasks" and "forgetting the date". In addition, the lower proficiency in basic smartphone operations and functions such as the calendar was also indicated in participants with SMCs, but it should be noted that the functions of smartphones are complex for middle-aged and older adults, and they may need assistance in dealing with SMCs by using their smartphones in their daily lives. When considering support for middle-aged and older adults in today's digital society, health professionals should focus on smartphone proficiency to address SMCs and build support for daily living.

## Appendices

**Table 5:** Questionnaire items of this study.

Scales	Response items
Age	
Sex	a. Male, b. Female
Education years	
Living situation	a. Living alone, b. Living with someone
Subjective memory complaints	
1. Do you forget the names of close friends or relatives?	a. Often, b. Occasionally, c. Not often, d. Not at all
2. Do you forget your appointment or task?	
3. Do you have difficulty remembering where you leave objects—like a wallet or a key?	
4. Do you have trouble figuring out what today’s date is?	
5. Do you forget what you want to buy at the supermarket or other places?	
6. Do you forget your way around the neighborhood?	
7. Do you consider yourself as being forgetful?	
Smartphone use and proficiency	
1. Do you use a smartphone?	a. Yes, b. No
MDPQ-16	
Navigate onscreen menus using the touchscreen	a. Never tried, b. Not at all, c. Not very easily, d. Somewhat easily, e. Very easily
Use the onscreen keyboard to type	
Send emails	
Send pictures by email	
Transfer information (files such as music, pictures, documents) on my mobile device to my computer	
Transfer information (files such as music, pictures, documents) on my computer to my mobile device	
Find information about my hobbies and interests on the Internet	
Find health information on the Internet	
Enter events and appointments into a calendar	
Check the date and time of upcoming and prior appointments	
Use the device’s online “store” to find games and other forms of entertainment (e.g. using Apple App Store or Google Play Store)	
Listen to music	
Setup a password to lock/unlock the device	
Erase all Internet browsing history and temporary files	
Update games and other applications	
Delete games and other applications	
